# Dilemmas in Endoscopic Management of Rectal Neuroendocrine Tumors: A Case-Based Discussion

**DOI:** 10.1155/2015/539861

**Published:** 2015-08-05

**Authors:** Brian P. Rajca, Mihir S. Wagh

**Affiliations:** Division of Gastroenterology, University of Florida, Gainesville, FL 32608, USA

## Abstract

Rectal neuroendocrine tumors are uncommon neoplasms that historically were regarded as having an indolent course. Due to the widespread use of screening colonoscopy neuroendocrine tumors of the rectum are identified with increasing frequency. More recent literature has suggested that rectal neuroendocrine tumors may progress in a more malignant fashion than previously believed. In this case-based discussion we present management dilemmas, analyze current guidelines, and highlight the role of endoscopic ultrasound, endoscopic resection, and surgery.

## 1. Introduction

The incidence of neuroendocrine tumors (NETs) of the colon and rectum has increased dramatically over the past several decades with a current incidence of approximately 1 per 100,000 [1, 2]. Although this increase was initially proposed to result from the widespread use of endoscopy for colorectal cancer screening the incidence of NETs of the stomach, pancreas, and small bowel has also multiplied for reasons that are not entirely clear [1, 3]. Historically the term carcinoid was used to differentiate the more indolent neuroendocrine tumors from the more malignant carcinomas of the gastrointestinal tract [4]. However, the World Health Organization (WHO) now recommends that the term carcinoid be reserved for only the most low grade tumors as many neuroendocrine tumors behave in a more malignant fashion than previously believed [5].

Neuroendocrine tumors (NETs) are derived from enterochromaffin cells which are located throughout the intestine within the crypts of Lieberkühn [4]. The development of neuroendocrine tumors typically occurs deep within these crypts and as these tumors progress they invade into the muscularis mucosa and the submucosa resulting in their subepithelial appearance [6]. Although patients with rectal NETs may present with nonspecific symptoms such as hematochezia, dyschezia, abdominal pain, or a change in bowel habits, nearly 50% of patients are asymptomatic and incidentally diagnosed during screening colonoscopy [7].

Historically, rectal NETs were regarded as indolent tumors with only a small number of patients developing complications from their disease. Recent studies, however, have suggested there exists a more considerable risk with rectal NETs and have found that regional metastasis may occur in as many as 5% of patients with subcentimeter lesions [8]. The 5-year survival rate approaches 85% for localized lesions and abruptly decreases to 50% for regional spread and 20–30% for distant metastasis [9, 10]. Although an 85% 5-year survival rate is high compared to other malignancies it is an unacceptably high rate for what is perceived by many physicians and patients to be a benign diagnosis. As a result of these and other studies the WHO now recommends that all rectal NETs be considered potentially malignant [5].

Guidelines from the North American Neuroendocrine Tumor Society (NANETS), the National Comprehensive Cancer Network (NCCN), and the American Joint Committee on Cancer (AJCC) provide guidance on the diagnosis, staging, and management of well differentiated neuroendocrine tumors (NETs) of the rectum [2, 11, 12]. Despite these guidelines our experience has identified several scenarios where management is difficult and not addressed by the guidelines. The aim of this review is to discuss these scenarios along with our approach to their management. We present a representative case that highlights these issues.

## 2. Case

**Figure 1:**
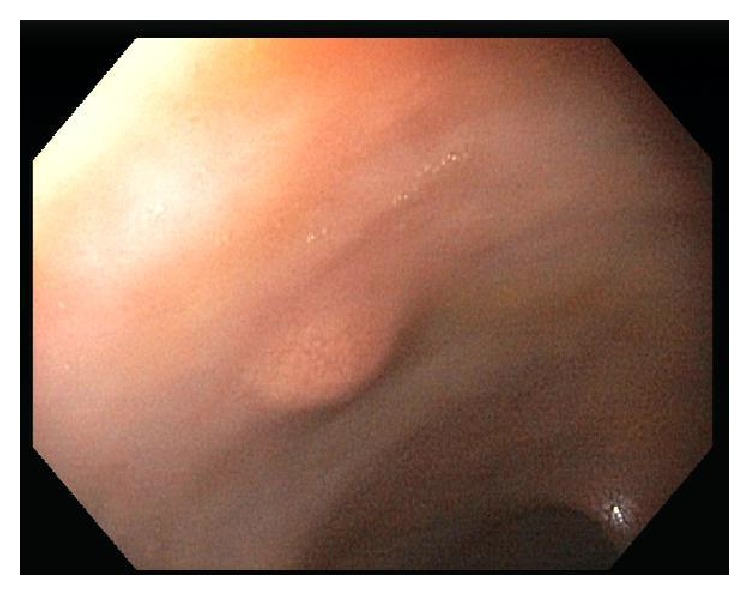
Rectal nodule on initial endoscopic examination.

**Figure 2:**
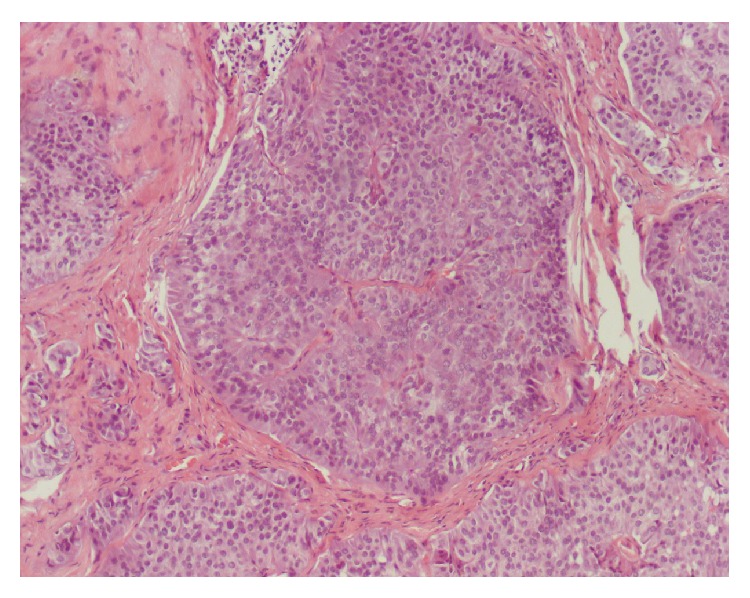
Resected specimen showing uniform neuroendocrine cells.

**Figure 3:**
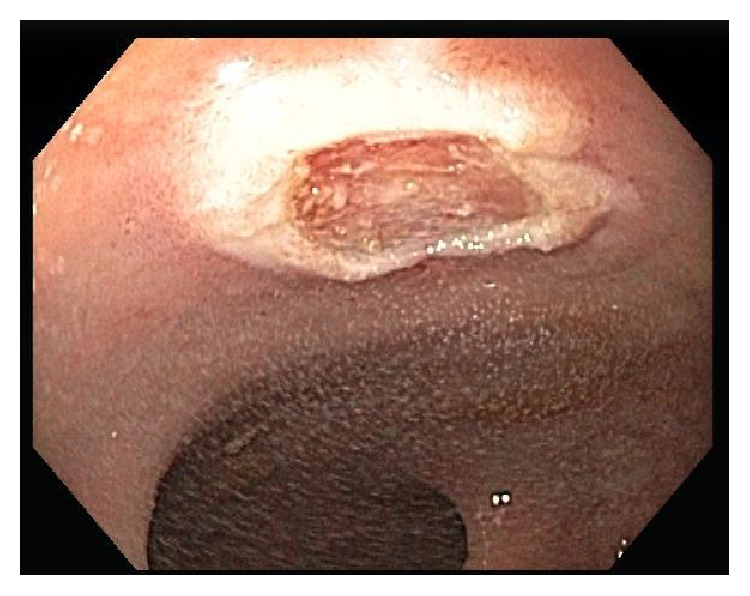
Neuroendocrine tumor after endoscopic mucosal resection.

A 68-year-old woman was referred for colorectal cancer screening. Her brother was diagnosed with colon cancer at age 80. She denied any gastrointestinal symptoms or change in weight and specifically denied diarrhea or flushing. Her past medical history was significant for allergic rhinitis and asthma and she had no prior surgical history. Her local gastroenterologist performed a screening colonoscopy which revealed a 6 mm rectal nodule which was biopsied (Figure 1). Pathology showed this to be a well differentiated rectal NET (Figure 2). A repeat flexible sigmoidoscopy was performed and the nodule in the rectum was resected and the site tattooed (Figure 3). An endoscopic ultrasound was not performed prior to this resection. Pathology again confirmed the lesion to be a well differentiated rectal NET. She was referred to our academic medical center where she underwent a rectal endoscopic ultrasound (EUS) which did not reveal a visible lesion or perirectal lymphadenopathy. The tattooed site appeared endoscopically normal but the biopsies surprisingly showed the presence of a well differentiated rectal neuroendocrine tumor with lymphovascular invasion.

## 3. Management Dilemmas

Guidelines for the management of rectal NETs have been established by the National Comprehensive Cancer Network (NCCN) and the North American Neuroendocrine Tumor Society (NANETS) [2, 11]. However, despite the guidelines there remain clinical scenarios where the management remains unclear. Our case illustrates a scenario that we have encountered on more than one occasion. Our patient had a nodule biopsied on the index colonoscopy consistent with a rectal NET, a scenario that is occurring with increased frequency with the widespread use of high definition colonoscopy [13]. At our index examination a visible lesion was not apparent endoscopically or on rectal endoscopic ultrasound; however the biopsies of the site revealed persistent disease.

**Figure 4:**
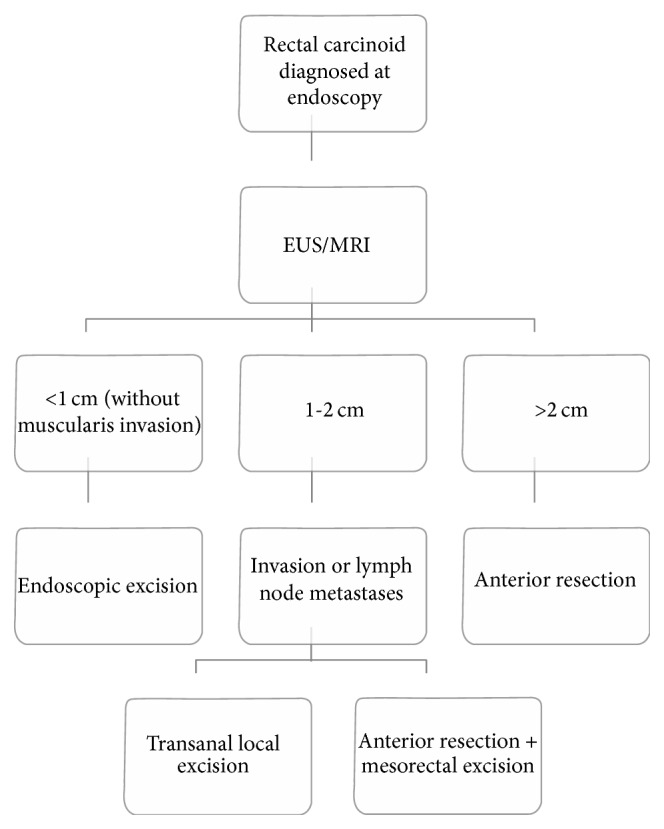
Management algorithm from the North American Neuroendocrine Tumor Society (NANETS) guidelines [2].

This scenario raises two main concerns, the first involving surveillance after resection. Although some recommendations address postresection surveillance they do not address all potential scenarios and vary depending on the organization. NANETS recommends against long term surveillance for stage 1 lesions which are defined as lesions ≤ 2 cm and not extending beyond the submucosa [2] (Figure 4). For stage II or III lesions (invading into or beyond the muscularis propria or with locoregional lymph nodes) NANETS recommend annual radiographic surveillance. Despite these recommendations the NANETS guidelines do not comment on the role of short term surveillance or endoscopic surveillance. Contrarily the NCCN does recommend short term endoscopic surveillance for lesions ≥ 1 cm in size at 6 and 12 months after resection with subsequent surveillance performed on an individualized basis. However, they do not explicitly recommend whether this should be performed with flexible endoscopy or with endoscopic ultrasound [11].

Both NANETS and NCCN are consistent in recommending against surveillance for lesions < 10 mm in size despite multiple studies showing that even low risk rectal NETs with complete resections and negative margins still carry a risk not only of local recurrence but also of distant metastatic disease [14, 15]. Soga reviewed 777 cases of rectal NETs and found the rate of metastasis from rectal NETs smaller than 10 mm to be 9.7% suggesting that even small rectal NETs carry a low risk of metastases at the time of diagnosis [16]. Similarly, Yamagishi et al. analyzed 20 patients who underwent surgical resection for rectal NETs and analyzed their resection specimens, specifically addressing the presence of metastases [17]. Metastatic disease was found at the time of resection in 3 of 5 (60%) of patients with tumors less than 10 mm in size. This study is limited by its size and showed a higher rate of metastasis than most previous studies but does have the strength of utilizing surgical resection specimens as the means of determining metastatic rates.

A recent retrospective review by Holinga et al. raised similar concerns regarding endoscopic surveillance [14]. These investigators analyzed 24 low risk patients with incidentally detected rectal NETs less than 10 mm in size and on surveillance found 2 of 24 patients (8.3%) had developed regional lymph node metastases detected by rectal EUS. In both cases a rectal EUS was performed at the time of the initial resection and revealed no evidence of metastatic disease. Both lesions were confirmed WHO Grade 1 lesions with Ki-67 staining ≤ 2% and without evidence of lymphovascular invasion on pathology confirming these to be low risk lesions. Although the guidelines suggest that surveillance endoscopy is not required in this situation a repeat rectal EUS performed in both patients (at 17 months and 26 months, resp.) after the initial diagnosis revealed evidence of perirectal lymph node metastases. Hence, there may be a role for EUS surveillance after resection but larger studies are needed to identify which patients may benefit from such an approach.

The rationale for recommending against routine surveillance for small lesions is based on high survival rates. The 7th edition of the AJCC TNM staging system for rectal NETs has been shown to reliably discriminate prognosis [12, 18]. Per AJCC TNM guidelines a patient with Stage I disease would have a 5-year survival of nearly 90% and hence surveillance is not recommended in the guidelines [12]. Although a survival rate of 90% is high there still is an overall 10% mortality rate at 5 years which may be unacceptably high for what has historically been considered an indolent neoplasm. We feel our patient would not be appropriate to consider disease-free and rather may be at an increased risk of disease recurrence and could benefit from surveillance after resection.

The second concern that our scenario raises is how to manage recurrent lesions, positive margins, or positive biopsies from nonvisible lesions. The guidelines are unclear in this setting. Since small rectal NETs carry a risk of disease progression and recurrence, it is concerning to find persistent disease on surveillance endoscopy [14, 15]. Multiple studies have suggested the presence of lymphovascular invasion and high mitotic rates are poor prognostic factors even with low risk rectal NETs but this has not been incorporated into the management algorithm [19, 20]. The European Neuroendocrine Tumors Society (ENETS), however, has incorporated the mitotic count and Ki-67 staining to determine histologic grade and does recommend a more aggressive resection or more aggressive surveillance for higher grade lesions [21].

It is likely that high mitotic counts, lymphovascular invasion, and high Ki-67 staining are features that may be present in low risk patients with small NETs that progress to develop recurrent disease after endoscopic resection. Although rectal NETs lacking these features may still carry a risk of metastasis these features may help predict which patients are at an increased risk of disease recurrence [8]. Several studies have investigated this issue but the studies are limited by small numbers and strong conclusions cannot be made [8, 16, 20]. Until larger studies are able to clarify this we believe patients with high mitotic counts, lymphovascular invasion, and Ki-67 staining may be the most likely to benefit from close endoscopic surveillance after endoscopic resection.

Our scenario also raises the possibility that patients with low risk lesions that progress may not have had complete resection and without surveillance endoscopy this may not have been detected. Despite having low risk lesions patients with persistent disease on a surveillance endoscopy or high risk features on pathology should be considered for a more aggressive resection such as a transanal excision or surgical resection. This is the approach recommended by ENETS but has yet to be fully incorporated into the US guidelines [21]. Our patient's residual lesion on biopsy makes it reasonable to consider additional surgical resection of the site.

Another management conundrum is where a rectal polyp is resected on routine colonoscopy but incidentally is found to be a rectal NET on pathology. In this setting, when the margins are negative the guidelines provide direction on surveillance. However, our experience has been that the resected margins are often positive which raises similar concerns as those represented by our case. In this setting the lesion has already been resected and may not be visible on future examinations making further management difficult. In addition an adequate staging EUS cannot be performed unless the original site of the resection can be identified by an endoscopic tattoo, residual tissue, or a postresection scar. These patients may benefit from close endoscopic surveillance and consideration of a more aggressive resection.

Another area of emphasis is the technique used for endoscopic resection. Many patients with a positive margin have undergone traditional endoscopic mucosal resection (EMR), often with snare polypectomy. Due to the submucosal nature of these lesions this technique does not allow for a deep resection and the deep margins may be positive. Several studies have compared EMR, cap-assisted EMR, and endoscopic submucosal dissection (ESD) for resection of rectal NETs [22–25]. A retrospective review of 115 patients undergoing resection of small (mean size 6.29 mm) rectal NETs found no difference in complication rates between EMR, cap-assisted EMR, or ESD but did find that complete histologic resection was more common with cap-assisted EMR or ESD (100% and 97.7%, resp.) as compared to EMR alone (77.4%) [22].

These results are corroborated by another retrospective study which found complete resection rates of 80% for lesions that underwent traditional EMR but 100% resection rates for lesions that were resected by cap-assisted EMR or ESD, though the study was small and likely underpowered [24]. Multiple head to head analyses of cap-assisted EMR and ESD have been performed and although there were no differences in completeness of the resection or complication rates, cap-assisted EMR was shown to have a shorter procedure duration when compared to ESD and thus may be the preferred technique [25, 26]. Although these studies are limited by their small size they have consistently shown lower rates of complete resection with traditional EMR. Given the limited availability of ESD and longer procedure times, cap-assisted EMR may be the preferred resection technique to ensure that negative margins are achieved as this is the first step in achieving disease-free survival. Both NANETS and the NCCN guidelines comment on when to consider endoscopic resection but they do not recommend the type of resection technique [2, 11]. Despite multiple studies showing that endoscopic submucosal dissection (ESD) or cap-assisted endoscopic mucosal resection (EMR-L) is superior to traditional endoscopic mucosal resection (EMR) this data has not been incorporated into the guidelines [22–25]. It is therefore advisable to avoid partial resection if technical expertise is not available at the time of the index endoscopy and to consider referral to a larger tertiary care center.

Our approach to rectal NETs < 10 mm is as follows: when a lesion suspicious for a rectal NET is incidentally encountered during colonoscopy we consider biopsies of the lesion and placement of an endoscopic tattoo prior to any resection. If the biopsies confirm the lesion is a rectal NET we proceed with a staging rectal EUS and also a MRI to check for evidence of metastasis. If pathology confirms a low grade lesion without lymphovascular invasion and there are no concerning features on EUS such as lymphadenopathy or involvement beyond the submucosa we typically proceed with endoscopic resection. If negative margins are achieved we follow the patient with close endoscopic surveillance with repeat EUS at 6–12 months. During surveillance we consider biopsies of the site even if a visible lesion is not seen. If the margins or surveillance biopsies are positive we will consider a transanal or surgical resection as needed. If there is no evidence of recurrence at 12 months, surveillance intervals are increased on an individualized basis.

## 4. Summary

Neuroendocrine tumors of the rectum are uncommon but are being identified with increasing frequency due to the widespread use of screening colonoscopy. Though recent guidelines have helped ensure a more standardized approach to these lesions they are not all inclusive. We believe that histologic features such as lymphovascular invasion, high mitotic rates, and high Ki-67 staining need to be considered as poor prognostic factors. Additional studies are needed to fully understand how strongly these factors contribute to disease recurrence in low risk rectal NETs but until then patients with these features may benefit from a more aggressive management approach such as surgical resection and closer endoscopic surveillance. Overall, rectal neuroendocrine tumors have a favorable prognosis when compared to traditional carcinomas of the colon and rectum. However, it is important to emphasize that there still exists a low but real risk of recurrent disease even when approaching low risk lesions.

## References

[1] Scherübl H. (2009). Rectal carcinoids are on the rise: early detection by screening endoscopy. *Endoscopy*.

[2] Anthony L. B., Strosberg J. R., Klimstra D. S. (2010). The NANETS consensus guidelines for the diagnosis and management of gastrointestinal neuroendocrine tumors (NETs): well-differentiated nets of the distal colon and rectum. *Pancreas*.

[3] Taghavi S., Jayarajan S. N., Powers B. D., Davey A., Willis A. I. (2013). Examining rectal carcinoids in the era of screening colonoscopy: a surveillance, epidemiology, and end results analysis. *Diseases of the Colon and Rectum*.

[4] Eggenberger J. C. (2011). Carcinoid and other neuroendocrine tumors of the colon and rectum. *Clinics in Colon and Rectal Surgery*.

[5] Bosman F. T., Carniero F., Hruban R., Thiese N. (2010). *WHO Classification of Tumours of the Digestive System*.

[6] Shimizu T., Tanaka S., Haruma K. (2000). Growth characteristics of rectal carcinoid tumors. *Oncology*.

[7] Wang A. Y., Ahmad N. A. (2006). Rectal carcinoids. *Current Opinion in Gastroenterology*.

[8] Kasuga A., Chino A., Uragami N. (2012). Treatment strategy for rectal carcinoids: a clinicopathological analysis of 229 cases at a single cancer institution. *Journal of Gastroenterology and Hepatology*.

[9] Modlin I. M., Lye K. D., Kidd M. (2003). A 5-decade analysis of 13,715 carcinoid tumors. *Cancer*.

[10] Weinstock B., Ward S. C., Harpaz N., Warner R. R. P., Itzkowitz S., Kim M. K. (2013). Clinical and prognostic features of rectal neuroendocrine tumors. *Neuroendocrinology*.

[11] Kulke M. H., Benson A. B., Bergsland E. (2012). Neuroendocrine tumors: clinical practice guidelines in oncology. *Journal of the National Comprehensive Cancer Network*.

[12] Edge S. B. (2010). *American Joint Committee on Cancer: Cancer Staging Manual*.

[13] Modlin I. M., Sandor A. (1997). An analysis of 8305 cases of carcinoid tumors. *Cancer*.

[14] Holinga J., Khalid A., Fasanella K., Sanders M., Davison J., McGrath K. (2012). Metastatic risk of diminutive rectal carcinoid tumors: a need for surveillance rectal ultrasound?. *Gastrointestinal Endoscopy*.

[15] Kobayashi K., Katsumata T., Yoshizawa S. (2005). Indications of endoscopic polypectomy for rectal carcinoid tumors and clinical usefulness of endoscopic ultrasonography. *Diseases of the Colon and Rectum*.

[16] Soga J. (2005). Early-stage carcinoids of the gastrointestinal tract: an analysis of 1914 reported cases. *Cancer*.

[17] Yamagishi D., Matsubara N., Noda M. (2012). Clinicopathological characteristics of rectal carcinoid patients undergoing surgical resection. *Oncology Letters*.

[18] Kim M. S., Hyuk H., Min B. S., Baik S. H., Lee K. Y., Kim N. K. (2013). Clinical outcomes for rectal carcinoid tumors according to a new (AJCC 7th edition) TNM staging system: a single institutional analysis of 122 patients. *Journal of Surgical Oncology*.

[19] Fahy B. N., Tang L. H., Klimstra D. (2007). Carcinoid of the rectum risk stratification (CaRRs): a strategy for preoperative outcome assessment. *Annals of Surgical Oncology*.

[20] Konishi T., Watanabe T., Kishimoto J., Kotake K., Muto T., Nagawa H. (2007). Prognosis and risk factors of metastasis in colorectal carcinoids: results of a nationwide registry over 15 years. *Gut*.

[21] Caplin M., Sundin A., Nillson O. (2012). ENETS Consensus Guidelines for the management of patients with digestive neuroendocrine neoplasms: colorectal neuroendocrine neoplasms. *Neuroendocrinology*.

[22] Kim K. M., Eo S. J., Shim S. G. (2013). Treatment outcomes according to endoscopic treatment modalities for rectal carcinoid tumors. *Clinics and Research in Hepatology and Gastroenterology*.

[23] Lee S. H., Ja Park S., Hun Kim H. (2012). Endoscopic resection for rectal carcinoid tumors: comparision of polypectomy and endoscopic submucosal resection with band ligation. *Clinical Endoscopy*.

[24] Zhao Z. F., Zhang N., Ma S. R. (2012). A comparative study on endoscopy treatment in rectal carcinoid tumors. *Surgical Laparoscopy Endoscopy & Percutaneous Techniques*.

[25] Niimi K., Goto O., Fujishiro M. (2012). Endoscopic mucosal resection with a ligation device or endoscopic submucosal dissection for rectal carcinoid tumors: an analysis of 24 consecutive cases. *Digestive Endoscopy*.

[26] Choi C. W., Kang D. H., Kim H. W. (2013). Comparison of endoscopic resection therapies for rectal carcinoid tumor: endoscopic submucosal dissection versus endoscopic mucosal resection using band ligation. *Journal of Clinical Gastroenterology*.

